# Hypothalamic Structure and Function in Alzheimer’s Disease and Lewy-Body Dementia: A Systematic Review and Meta-Analysis

**DOI:** 10.1192/bjo.2025.10097

**Published:** 2025-06-20

**Authors:** Axel AS Laurell, Sita N Shah, Masoud Rahmati, John T O’Brien, Benjamin R Underwood

**Affiliations:** ^1^University of Cambridge, Cambridge, United Kingdom; ^2^Cambridgeshire and Peterborough NHS Foundation Trust, Cambridge, United Kingdom; ^3^Aix-Marseille University, Marseille, France; ^4^Lorestan University, Khoramabad, Iran, Islamic Republic of; ^5^Vali-E-Asr University of Rafsanjan, Rafsanjan, Iran, Islamic Republic of

## Abstract

**Aims:** Changes to sleep, weight, and endocrine function are common in Alzheimer’s disease (AD) and Lewy-body dementia (LBD). The cause of these is not known, but they may be related to hypothalamic neurodegeneration. Our aim was to assess whether hypothalamic volume is reduced in people with AD and LBD, and whether hypothalamic volume is associated with these common symptoms.

**Methods:** We performed a systematic search of MEDLINE and EMBASE for studies using structural magnetic resonance imaging to examine hypothalamic volume in AD or LBD. The Newcastle–Ottawa scale was used to assess the risk of bias. A random-effects meta-analysis was conducted using the standardised mean difference (SMD) in hypothalamic volume, and a narrative synthesis was used to examine the relationship between hypothalamic volume and sleep, weight, and endocrine function.

**Results:** We screened 6542 articles which identified 12 studies for inclusion, of which 10 had a low to moderate risk of bias. People with mild-moderate AD had a significantly smaller hypothalamus (−10.1%) compared with controls (pooled SMD= −0.49 (−0.86 to −0.13), p=0.018; I^2^=67% (21.5–86.1%); n= 454 (AD), 715 (controls)). The only study in people with LBD found grey matter loss in the hypothalamus compared with controls using voxel-based morphometry. Hypothalamic volume loss in AD was more marked in men and was associated with plasma levels of sex hormones and reduced bone mineral density. Body mass index, appetite and sleep were not associated with hypothalamic volume in AD.

**Conclusion:** Reduced hypothalamic volume is seen early in AD and this may influence endocrine function. A better understanding of hypothalamic degeneration in dementia may help elucidate how pathology relates to symptoms in AD and LBD and reveal new targets for intervention.

